# Visual motion integration of bidirectional transparent motion in mouse opto-locomotor reflexes

**DOI:** 10.1038/s41598-021-89974-y

**Published:** 2021-05-18

**Authors:** L. A. M. H. Kirkels, W. Zhang, Z. Rezvani, R. J. A. van Wezel, M. M. van Wanrooij

**Affiliations:** 1 Department of Biophysics, Donders Institute, Radboud University, Nijmegen, The Netherlands; 2 School of Computer Science, Institute for Research in Fundamental Sciences, Tehran, Iran; 3 Biomedical Signals and Systems, TechMed Centre, Twente University, Enschede, The Netherlands

**Keywords:** Oculomotor system, Motion detection, Decision, Perception, Behavioural methods, Psychophysics

## Abstract

Visual motion perception depends on readout of direction selective sensors. We investigated in mice whether the response to bidirectional transparent motion, activating oppositely tuned sensors, reflects integration (averaging) or winner-take-all (mutual inhibition) mechanisms. We measured whole body opto-locomotor reflexes (OLRs) to bidirectional oppositely moving random dot patterns (leftward and rightward) and compared the response to predictions based on responses to unidirectional motion (leftward or rightward). In addition, responses were compared to stimulation with stationary patterns. When comparing OLRs to bidirectional and unidirectional conditions, we found that the OLR to bidirectional motion best fits an averaging model. These results reflect integration mechanisms in neural responses to contradicting sensory evidence as has been documented for other sensory and motor domains.

## Introduction

The brain receives visual motion information about moving objects and motion patterns induced by self-motion via direction-selective neurons in cortical and subcortical brain areas. Subsequently, the activity of these motion sensors has to be translated into a motion percept and/or appropriate motor behaviour. Although the visual motion system has been studied extensively for decades, the exact mechanisms by which activity of motion sensors tuned to different directions is read out for perception or for driving motor actions is still under discussion^1–5^. Typically, only one motor-output direction is possible in reflexive eye, head or whole-body movement responses driven by large-field moving patterns. When these body movements are driven by two simultaneously presented visual-motion patterns moving in opposite directions (bidirectional motion), there are two basic readout mechanisms possible: integration or winner-take-all (WTA). For integration, activity of motion sensors tuned to different motion directions is combined into one vector, which we will refer to as averaging. The WTA combination rule allows for mutual inhibition between oppositely tuned motion sensors, and eventually only the most prevalent stimulus will drive the output^1,5^.

**Figure 1:**
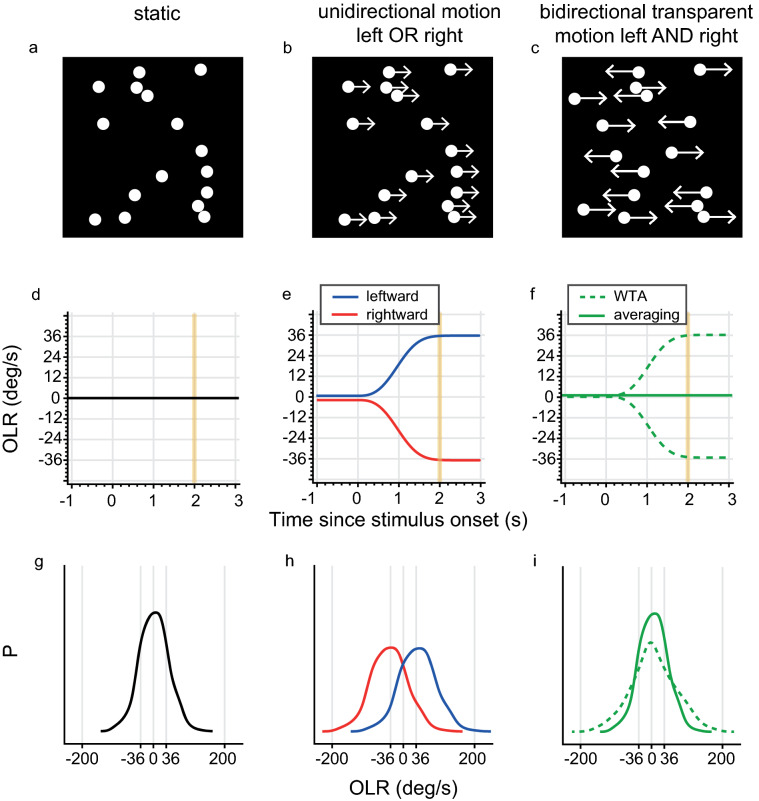
Stimuli and rationale. (**a**–**c**) Schematic drawings of the (**a**) static, (**b**) unidirectional right and (**c**) bidirectional visual motion stimuli. (**d**–**f**) Ideal schematic OLRs over time for the (**d**) static, (**e**) unidirectional and (**f**) bidirectional stimuli are shown, without response variability, biases or outliers. (**e**) Responses to left or right unidirectional motion are indicated by blue and red, respectively. (**f**) Note that the two models yield different responses: the averaging model, indicated by the solid green line, predicts an OLR of 0, while the WTA model, indicated by the dashed green line, predicts either a leftward or rightward turn. The vertical yellow lines indicate end of stimulus, 2 s after stimulus onset. (**g**–**i**) Schematic OLR probability distribution determined at 2 s after stimulus onset are shown for each stimulus, including response variability. The drawings for the (**g**) static and (**h**) unidirectional stimuli are fictional, but resemble data observed in earlier reports^18,19^. (**i**) The OLR probability distributions according to the WTA (dashed green line) and averaging (solid green line) readout rules are predicted from the fictional unidirectional motion response data in (**h**) (see Methods for exact predictions determined from unimodal response distributions). Note that both distributions are unimodally peaked, yet can be distinguished by their width.

To study readout mechanisms in visual motion processing, the response to two simultaneously moving patterns in different directions is measured^6–10^. Here, we used bidirectional random dot patterns which are created from two populations of superimposed dots that move in different directions^8,11–17^ (Fig. 1a–c). By comparing one versus two simultaneously presented stimuli, we aim to determine how the response to two simultaneous stimuli relates to the response to the separate components.

In previous work^18^ we developed a method to measure reflexive turning of running responses of mice to moving random dot patterns, called opto-locomotor reflexes (OLRs). In these measurements, mice are head-fixed and running on an air-floating Styrofoam ball and stimulated with moving stimuli covering a large part of the visual field. The onset of such a large-field moving stimulus evokes a reflexive compensatory response of the mouse by running in the same direction as the motion stimulus. In this study, we are interested in how mice process motion information of bidirectional random dot patterns moving in opposite directions in order to test what readout rule applies for the OLR.

Each readout rule leads to different predictions of the OLR to the various presented dot patterns (see Fig. 1 for rationale). When the pattern is stationary (Fig. 1a), the mouse is expected to run straightforward (Fig. 1d) for either readout rule. Because in the opto-locomotor setup the mouse can choose freely to run in every direction, variability across actual responses can be quite large in practice (Fig. 1g). When the pattern is moving in one direction (unidirectional, Fig. 1b), the mouse is expected to run in the same direction as the moving pattern (Fig. 1e) again for both readout rules, but again, in practice, actual response variability across trials can be large as the mouse is free to move (Fig. 1h).

Only when two oppositely moving random dot patterns are presented simultaneously, we can differentiate between the averaging and WTA rules (Fig. 1c). Ideally, both models yield extinguishable traces, with an OLR of 0 for averaging and either leftward or rightward turns for WTA (Fig. 1f). However, in practice variability across actual responses can be quite large and the average of trials following WTA readout rules could be indiscernible from trials following averaging. However, the models could then still be distinguished based on their response distributions. We randomly sampled from unidirectional data to left and right stimulation to derive OLR probability distributions for bidirectional motion (Fig. 1i, see Methods for exact predictions).

The averaging rule (Fig. 1i, solid green line) will produce a sharply-peaked unimodal distribution, (as it is a distribution of the average of two independently distributed variables, Methods Eq. 4). The WTA readout rule (Fig. 1i, dashed green line) will consist of an equal mixture of leftward and rightward response distributions, (i.e., a mixture distribution of two independently distributed variables, Methods Eq. 2). Depending on the size of the response variability, this distribution will be bimodally peaked with either mode at the response produced by either the left or right unidirectional-motion stimulus (not shown); or, if the opto-locomotor reflex variability is large (compared to the differences in the means of the subpopulations), the WTA distribution will have a single wide peak with the mode at the average (Fig. 1i, dashed green line). In either case, there will be no difference between the ‘average’ response for the WTA and averaging rules, but the variance of the predictions will be different (Fig. 1i, dashed green line versus solid green line).

## Results

We recorded opto-locomotor reflexes (OLRs)^18^ of five mice in response to sudden onset of moving dot patterns. The stimuli consisted of four different motion types: unidirectional (horizontally leftward or rightward), static, and bidirectional motion (Fig. 1a–c). In the bidirectional motion stimulus two random dot patterns were superimposed and moving in opposite directions (horizontally moving leftward and rightward). The stimuli were presented to both eyes.

**Figure 2:**
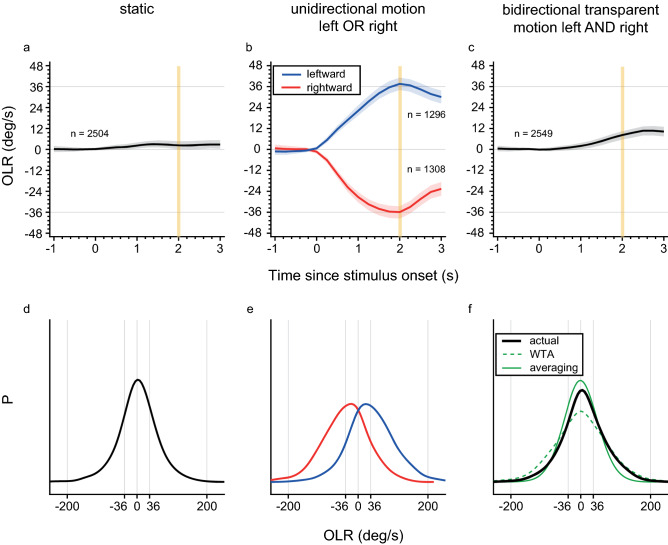
OLR mean and probability distributions. (**a**–**c**) Mean OLRs over time averaged across trials of five mice for the (**a**) static, (**b**) unidirectional left (blue) or right (red) and (**c**) bidirectional visual motion stimuli. Shaded areas represent 95% confidence intervals of the mean across trials. Trial numbers are depicted in each panel. The vertical yellow lines indicate end of stimulus, 2 s after stimulus onset. (**d**–**f**) OLR probability distributions determined at 2 s after stimulus onset are shown for each stimulus, including response variability. (**f**) The OLR probability distributions according to the WTA (dashed green line) and averaging (solid green line) readout rules are predicted from the actual unidirectional motion response data in (**e**). The actual OLR probability distribution is represented by the solid black line.

In Fig. 2a–c the average OLR for five mice are depicted in response to the different types of motion stimuli. Stimulation with a static pattern resulted in an average OLR where mice did not move to either side (Fig. 2a; note that the mice did run, on average, straightforward in the pitch direction, for which the data is not shown, but there is on average no sideways running). For unidirectional motion (left or right), mice turned on average in the same direction as the motion direction of the dot patterns (Fig. 2b, blue and red), corresponding to our previous findings^18–20^. In the bidirectional-motion stimulus condition, two dot patterns moving in opposite directions (leftward and rightward) were superimposed and presented to both eyes. This bidirectional-motion stimulus led to an OLR that was not completely straight, but showed some bias toward the left (Fig. 2c), as also reflected in a shift of the peak of the distributions in Fig. 2f. This leftward bias for bidirectional motion was prominent in two individual mice, M1 and M4 (see Supplementary Fig. S1), but was not present in the other three.

We plotted the probability distributions for the OLR at stimulus offset (2 s after stimulus onset) to static and unidirectional motion (Figs. 2d, e), where the largest difference of the OLR to rightward and leftward motion is observed. The distributions are wide, corresponding to a high variability in OLRs, probably due to the fact that mice can run freely on the ball without any training to run in a certain direction. The response distribution to bidirectional motion (Fig. 2f, solid black line) is unimodal, centred around 0. In this figure, we also included predictions for the averaging (solid green line) and WTA (dashed green line) readout rules, calculated from the unidirectional data (see Methods). The measured response distribution to bidirectional motion at stimulus offset (Fig. 2f, solid black line) highly resembles the averaging-rule prediction (Fig. 2f, green solid line), with a high peak probability around 0 deg/s and a relatively narrow distribution. These distributions have also been determined for each individual mouse (see Supplementary Fig. S1) and at least for three of the mice the averaging rule was clearly favoured; for the other two (M1 and M4) the distinction between the WTA and averaging rules was small.

**Figure 3:**
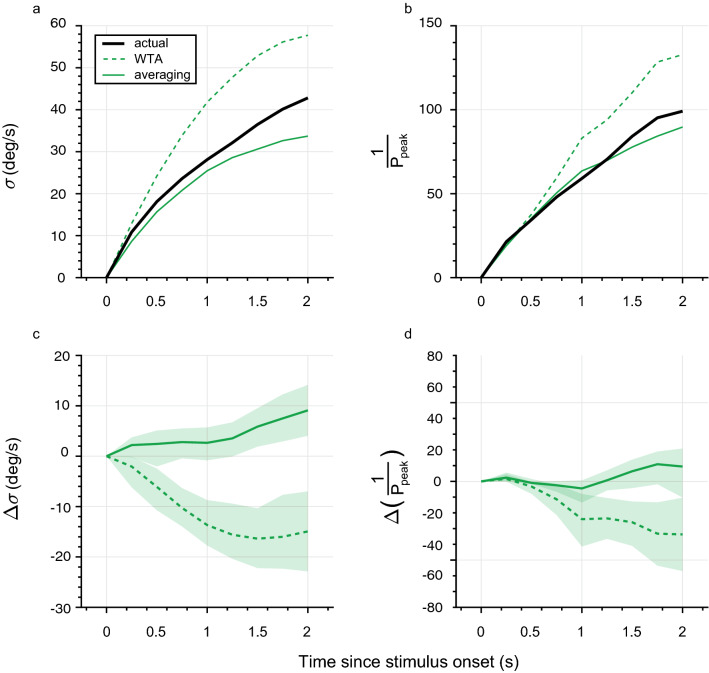
Temporal characteristics. (**a**) Averaged standard deviation of measured OLRs to bidirectional transparent motion (actual; solid black line) compared to that of WTA (dashed green line) and averaging (solid green line) predictions. (**b**) Inverse of the peak probability of measured OLR data (actual; solid black line) compared to that of WTA (dashed green line) and averaging (solid green line) predictions. (**c**) Difference in average standard deviation between measured OLR and WTA (dashed green line) or averaging (solid green line) predictions. (**d**) Difference in inverse peak probability between measured OLR and WTA (dashed green line) or averaging (solid green line) predictions. All curves are averages across all animals (*n* = 5). Shaded areas represent 95% confidence intervals of the mean across animals (*n* = 5).

To visualize and quantify this for the entire course of the OLR during stimulation, we compared both the standard deviation and the (inverse of the) peak probability of the actual response distributions to those of the WTA and averaging predictions over time (Fig. 3). The standard deviation of the actual OLRs gradually and monotonically increases over time (Fig. 3a, black straight line) to a standard deviation of about 36 deg/s larger than at stimulus onset. This indicates that the mice start changing their movement direction at time t = 0 s, and reach their maximum velocity at stimulus offset (t = 2 s); the magnitude and direction of the movements, however, are basically random, so that no consistent average is observed, except for a small leftward bias (Fig. 2c). The averaging prediction (Fig. 3a, solid green line) exhibits the same trend, although the predicted standard deviations are smaller than the actual values, by about 6 deg/s at stimulus offset. The WTA rule predicts much larger standard deviations, especially at stimulus offset (~ 55 deg/s, Fig. 3a). At first glance, the course of the OLR standard deviation seems to correspond mostly to the averaging prediction. This is also verified by the difference in predicted and measured values (Fig. 3c). Both predictions do not match the actual response exactly, but the WTA rule seems to deviate more.

Additionally, we quantified how the inverse of the peak probability changed over time (Fig. 3b). For a normal distribution, the inverse of the peak probability and the standard deviation are strictly related and should not yield any different conclusions. For a non-normal distribution, however, differences may occur, for example if the distribution contains extreme outliers (to which the standard deviation is more sensitive). For this measure, the difference between actual data and the averaging prediction is very small (Fig. 3d, solid green line; for most time points, the 95% confidence interval includes 0 deg/s), while the difference to the WTA prediction remained substantial (Fig. 3d, dashed green line). Overall, the results show that the OLR to bidirectional motion is best predicted by the averaging readout rule.

## Discussion

In order to investigate the readout mechanism of large-field motion-induced optokinetic reflexes we measured mouse opto-locomotor reflexes (OLRs) to bidirectional motion. The OLRs were compared to predicted OLR behaviour based on a combination of the responses to unidirectional motion. We compared two models: averaging and winner-take-all (WTA) and their temporal characteristics. These measurements were done under binocular conditions.

In our experiment we found that in response to bidirectional motion mice on average ran straight with a small bias toward the left. Possible explanations for this bias will be discussed later. The underlying distribution of OLRs to bidirectional motion at 2 s after stimulus onset showed a unimodal distribution that was similar to that for static stimuli. Although such a unimodal distribution can be explained by both the WTA and averaging model, we found that our measured OLR distribution most resembled that of averaging. Furthermore, a more quantitative approach comparing both models to the measured data substantiated this finding, as averaging predictions showed the smallest differences with the actual data. The finding that an averaging readout model best describes the OLR to bidirectional motion, is very characteristic for activity of midbrain structure superior colliculus (SC), which integrates input of multiple sensory modalities^21–24^ and is essentially involved in target selection for either orienting eye movements^25–33^, head and body movements^34–39^, or limb movement^40–42^. Moreover, direction sensitive neurons in the SC of mice are strongly biased for temporonasal motion^43^, suggesting an important role for SC in the asymmetry found in the OLR as well^19^. Taken together, this supports our notion that the SC is involved in mouse reflexive running behaviour and provides an integrative response, mirrored by the averaging seen in the OLR.

We find a leftward bias in response to bidirectional motion under binocular conditions. Although this bias seems more prominent in two individual subjects (see Supplementary Fig. S1), in general we do see larger responses combined with wider response distributions for left eye stimulation. This might reflect a bias for information retrieved from the left eye, or an effect from our setup. The ball might be easier to turn toward the left than the right because of mechanistic flaws in the setup. Alternatively, it is possible that the positioning of head plates is not symmetric and as a consequence thereof the mice run a bit askew on the ball which leads to a running direction bias. However, for all these explanations it is not clear why the mice have no bias in their running behaviour in the control condition with presentation of stationary dot patterns.

Note that two mice (M1 and M4) had clear leftward biases (see Supplementary Fig. S1), while the other three had negligible biases. For the three with less biases, the averaging scheme resembled the data best. For the two mice with biases, the distinction between both models was less. So, although WTA might be slightly favoured in these two mice, the averaging scheme might still be a possibility. Taken together with the pooled results (Fig. 2) and comparison across animals (Fig. 3), we would still argue that averaging is the most likely readout rule.

The fact that in our experiment mice do not choose one of the two motion directions for the bidirectional motion stimulus (Figs. 2f and 3), does not necessarily imply that mice cannot perceive the two motion directions. From a survival point of view, it is clear that distinguishing motion of occlusive or bidirectional objects and shadows from motion of the background can be of vital importance for organisms. Humans are able to see two bidirectionally moving patterns as separate moving patterns, and also in the setup that we used the two motion directions are discernable for humans. On the other hand, studies have shown that under bidirectional conditions interactions occur e.g.^17^. Furthermore, there are conditions where motion information is fully integrated in human perception, such as during the motion aftereffect of opponent bidirectional motion, where humans do not report any motion aftereffect at all^17,44–46^. From neurophysiological studies in primary visual cortex and extrastriate motion sensitive areas in cats and macaques we know that the activity is suppressed under bidirectional motion conditions and more suppressed the higher in the cortical hierarchy^16,47–49^. If we assume that subcortical mechanisms are underlying the OLR, our results suggest that motion signals are fully subtracted in subcortical motion processing areas. From our results one could argue that the OLR can be described by a fully opponent classical extended Reichardt detector^50^ or motion energy models^51^ for motion processing.

The running response to visual motion in the OLR is to a certain extent comparable to eye movements induced by large moving patterns. For eye movements it is well established that the superior colliculus first integrates visual input before selecting a target via WTA behaviour^52,53^, resulting in one object to make a saccade to^54–57^. An alternative consideration is the opposite order of events, where the OLR would first shows WTA behaviour which is followed by averaging, reflecting the sequence of segregation and integration mechanisms seen in some other sensory domains in response to contradicting sensory evidence^58^. Both considerations, however, do not fit our results, because we only observe averaging of the response during the whole stimulation period.

We expect that the averaging readout that we observe in the OLR is the result of subcortical processes, but cortical processes cannot be excluded. Previous literature remains inconclusive concerning integration and segregation of visual motion processing in mouse cortical areas and often compare local motion versus global motion responses of neurons that are referred to as component versus pattern (or plaid) cells. Findings on component and pattern cells in primary visual cortex (V1) and extrastriate areas in the mouse implicate that area V1 is more dedicated to local motion processing with more component cells^59^, which is comparable to motion processing in area V1 of higher mammals^60,61^. Conversely, another study investigating local integration in mouse visual cortex, argued that pattern selectivity is present in V1^62^. Finally, comparing mouse area V1 to primate and cat V1, Palagina et al.^63^ found that the proportion of component cells is considerably smaller, and the proportion of pattern cells is larger in mouse V1 than in primate or cat V1. Moreover, results from their V1 lesion studies suggest that area V1 contributes to complex motion perception. This supports a more prominent role of V1 in motion integration in mice compared to primates. Therefore, it is possible that the averaging readout we find in the OLRs is the result of integration mechanisms in striate and extrastriate cortical areas in mice. Future lesion studies and neural recordings combined with different parameter settings might provide a more comprehensive understanding of the underlying mechanisms of the OLR and involvement of subcortical and cortical areas.

## Conclusion

Opto-locomotor reflexes to bidirectional motion in freely-running mice is best described by averaging of two oppositely-directed signals, which seems in line with how sensorimotor maps, such as superior colliculus, are thought to encode a single goal-oriented response based on conflicting inputs.

## Methods

### Animals

Five male C57BL/6 J mice (6–8 weeks old) were used in this experiment. After a habituation period of 1 week, during which the animals got accustomed to their experimenter and setup, the mice went on food restriction and were each fed 2.2 g per day. The weight of the mice was maintained between 85 and 95% of their initial weight (22.8 ± 0.3 g). The mice were kept on a 12 h dark/12 h light cycle and all measurements took place during the dark cycle. All experiments were conducted in compliance with Dutch and European laws and regulations and were approved by the animal ethical committee of Radboud University Nijmegen. This study was carried out in compliance with the ARRIVE guidelines for animals.

### Surgery

Mice were anaesthetised via a nose cone (induction with 4% isoflurane in oxygen, kept at 1.0–1.5% during surgery, 0.2L/min). The mice were immobilised in a stereotactic holder and an antibacterial ophthalmic ointment (Puralube, Dechra) was applied to the eyes to prevent dehydration of the eyes. Subsequently, the heads of the mice were shaven and an incision was made in the skin to expose the skull. The exposed periosteum was treated with a local anaesthetic compound (1 mg/ml Lidocaine HCL with 0.25 mg/ml Bupivacain Actavis), before the skull was cleaned using a bone scraper. A custom-made titanium head-plate was attached to the skull with dental cement (SuperBond C&B, Sun Medical), allowing fixation of the head.

### Visual stimulation

**Figure 4:**
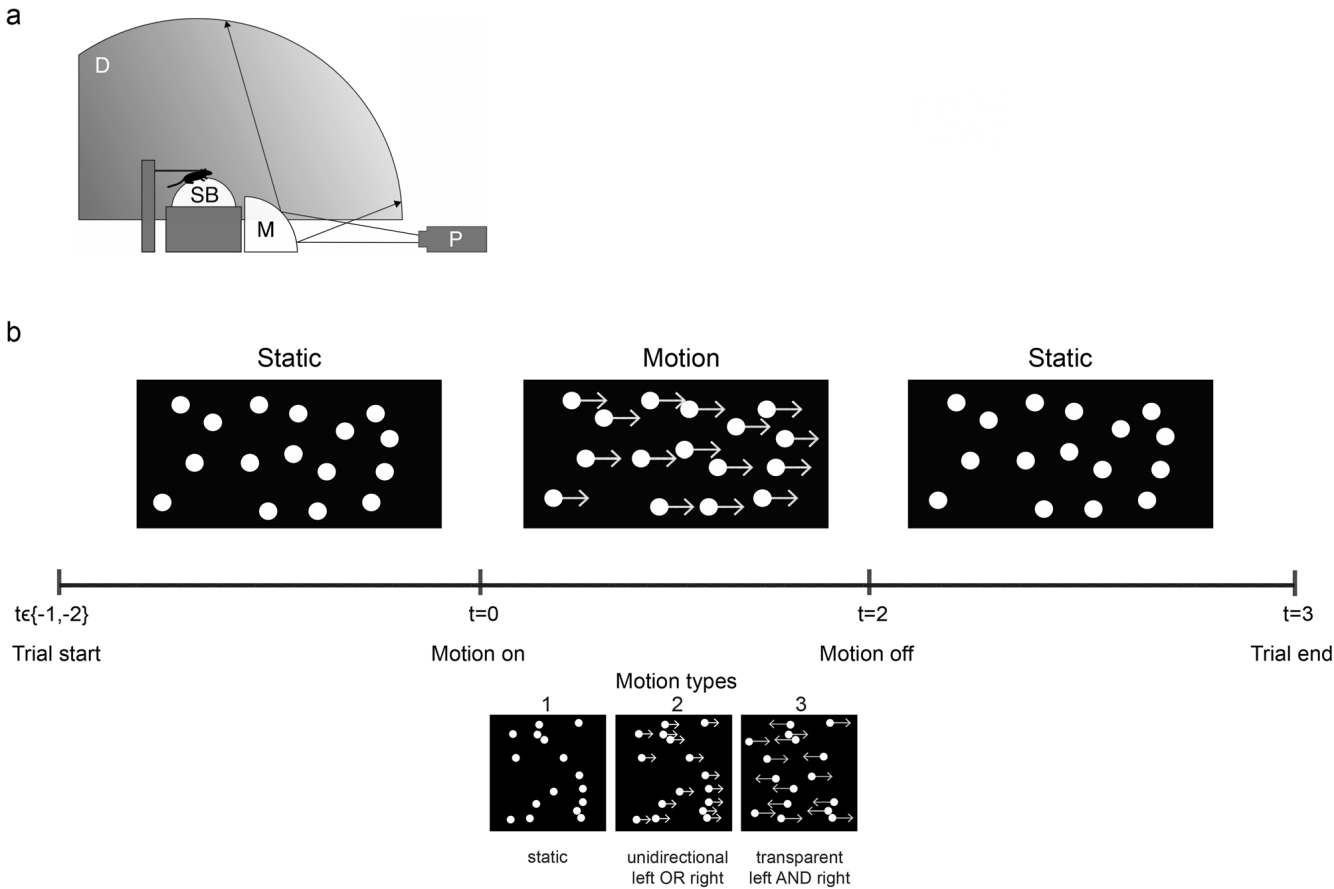
Experimental setup. (**a**) Schematic drawing of the setup. Projector (P) displayed patterns of randomly positioned dots via a mirror (M) onto the inside of a dome (D). Mice ran under head-fixed conditions on a Styrofoam ball (SB) floating on air. (**b**) The time course of trials for (moving) dot patterns. Trials started with presentation of either 1 or 2 s of stationary dots followed by 2 s of one of four “motion types” (1) static, (2) unidirectional leftward OR rightward moving at 36 deg/s or (3) bidirectional transparent leftward AND rightward moving at 36 deg/s. Trials ended with 1 s of stationary dots.

Our setup consisted of a Styrofoam ball (19.7 cm diameter, 48.5 g) that was floating on pressurised air in a semi-spherical socket^64^, adapted from insect studies^65–67^ (Fig. 4a). Mice were positioned on the ball using two metal bolts that connected the head plate to make sure the mice were held on the upper surface of the ball. On the inside of a spherical screen (inner diameter 112 cm), made of fiberglass-reinforced resin (Fibresports UK; Basildon, UK), an Optoma X501 video projector (1920 × 1080 resolution, 60 Hz) projected random dot patterns moving at a speed of 36 degrees per second. Horizontally, the visual stimuli covered 220 degrees of visual angle and vertically from 10 degrees below the mouse to 80 degrees above it (for details see Kirkels et al.^18^).

Stimuli consisted of patterns of white dots (0.20 cd/m^2^), with a radius of 1.4 degrees on a black background (0.09 cd/m^2^) randomly positioned in the stimulus window, with unlimited lifetime. The beginning of a trial started with randomly positioned static dots that started moving either 1 or 2 s after onset of the trial, to reduce effects of anticipation of the stimulus onset. Motion was generated by adjustment of azimuth and zenith of the dots each video frame (every 17 ms) and lasted 2 s. After 2 s of stimulus motion, the trial ended with a static dot pattern for 1 s (Fig. 4b). The dots were displaced such that their displacement resembled leftward or rightward unidirectional motion, bidirectional motion or they remained static. In unidirectional motion, all dots moved with an unlimited lifetime of the dots in the same direction (either left or right) at a speed of 36 deg/s. Bidirectional motion was created by two superimposed dot patterns moving in opposite directions (left and right) at the same speed (36 deg/s). Additionally, we also measured responses to random motion (motion energy in all directions) and conducted all experiments also under monocular conditions. These data are available upon request.

### Behavioural paradigm

After the habituation period, experimental sessions began. Twice daily, mice performed a 40-min experiment with two sessions of approximately 20 min. In total, 7 sessions per eye condition per mouse were recorded. In one session five different motion types were pseudorandomly presented (left unidirectional, right unidirectional, bidirectional, random motion and static). In one session the unidirectionally left and rightward moving stimulus was repeated 40 times each, the bidirectional condition 80 times, the random motion condition 80 times and the stationary random dot stimulus was repeated 80 times. For further analysis, we will not discuss random motion data and monocular conditions and focus on binocular stimulation for static, unidirectional left or right and bidirectional motion (240 trials per session). This resulted for three mice in 1680 trials (7 sessions × 240 trials). One mouse only finished 6 sessions, another mouse contributed 8 sessions. We excluded those trials in which the mice were sitting still by requiring that the standard deviation of both the yaw and pitch speeds over the course of the trial exceeded 5 deg/s. Total trial numbers are depicted in Fig. 2, individual trial numbers can be found in Supplementary Fig. S1.

### Recording and data analysis

We used two optical computer mice, one located in front and one to the side of the ball, to register yaw in deg/s with a sampling rate of 60 Hz. The yaw is considered a proxy for OLR of mice, since the OLR is transferred to the ball resulting in a rotation around the yaw axis. Every trial a baseline response was determined by taking the average yaw during 500 ms before stimulus onset. Subsequently, this baseline was subtracted from the yaw-over-time traces. A 100-ms boxcar filter was applied to each trace for all trials. Per mouse, mean traces were calculated and pooled over sessions. For describing the course of the (difference in) standard deviation and inverse of the peak, we normalized to the values at the moment of stimulus onset (t = 0 s). The 95% CIs of the mean are calculated via bootstrapping of the data, for the average difference (Fig. 3) this is over all animals (*n* = 5), for the mean OLR data this is over all trials (see trial numbers Fig. 2).

### Predictions

To clarify how the WTA and averaging readout rules differ for a response *X*, we can determine the expected or average response, *E*[*X*], and response variance, *E*[(*X* – *µ*)^2^]. For the WTA model this is as follows:

1 $$E\left[ X \right] = \mu_{b} = \frac{1}{n}\mathop \sum \limits_{u = 1}^{n} \mu_{u} = 0$$

2 $$E\left[ {\left( {X - \mu } \right)^{2} } \right] = \sigma_{b}^{ 2} = \frac{1}{n}\mathop \sum \limits_{u = 1}^{n} \left( {\sigma_{u}^{ 2} + \mu_{u}^{ 2} } \right)$$

where *n* = 2 for bidirectional motion and *µ*_*b*_ refers to the mean of responses to bidirectional motion, while *σ*_*u*_ indicates the standard deviation of responses to unidirectional motion.

For the averaging rule (a distribution of average of two normally-distributed independent variables) the average and variance are as follows:

3 $$E\left[ X \right] = \mu_{b} = \frac{1}{n}\mathop \sum \limits_{u = 1}^{n} \mu_{u} = 0$$

4 $$E\left[ {\left( {X - \mu } \right)^{2} } \right] = \sigma_{b}^{ 2} = \frac{1}{n}\mathop \sum \limits_{u = 1}^{n} \sigma_{u}^{ 2}$$

Note that for the averaging rule the standard deviation of the bidirectional motion response distribution is smaller than for either unidirectional response distributions (Eq. 4). Note, only the variance (or standard deviation) is different for both models when the mean response is large enough. The average, however, is the same for WTA and averaging.

To calculate the predictions of the responses to bidirectional motion based on the responses to unidirectional motion we used bootstrapping to randomly pick 10,000 samples (with replacement) from our unidirectional response data to left and right stimulation each. Subsequently, we applied each readout rule. For the averaging rule, the two sampled data streams are averaged. For the WTA rule, either the left or right sample was randomly picked with equal probability. From this sampled data, we determined the probability distributions and their standard deviation.

## Supplementary Information

Supplementary Information 1.

## Data Availability

The datasets generated during and/or analyzed during the current study are available in the Donders Repository, https://data.donders.ru.nl/collections/di/dcn/DSC_62001435_01_530.
