# Supplementary material for: Visual motion integration of bidirectional transparent motion in mouse opto-locomotor reflexes
Source: Sci Rep. 2021 May 18;11:10490. doi: 10.1038/s41598-021-89974-y (PMC8131598; doi:10.1038/s41598-021-89974-y)
Supplement: Supplementary file 1 — Supplementary Information 1. [file 41598_2021_89974_MOESM1_ESM.docx]

Visual motion integration of bidirectional transparent motion in mouse opto-locomotor reflexes

Kirkels, L. A. M. H., Zhang, W., Rezvani, Z., van Wezel, R.J.A., van Wanrooij, M.M.

**Fig. S1.** OLR mean and probability distributions for individual animals. Mean OLRs over time averaged across trials (upper panels) and OLR probability distributions (lower panels) per animal (M1–5) are shown in response to binocular stimulation with different visual motion stimuli (static, unidirectional left or right and bidirectional). Shaded areas represent 95% confidence intervals of the mean across trials. Trial numbers are depicted in each panel. The vertical yellow lines indicate end of stimulus, two seconds after stimulus onset. The OLR probability distributions are determined at two seconds after stimulus onset. The lower right panel shows the predicted OLR probability distributions for bidirectional motion according to WTA (dashed green line) and averaging rules (solid green line) rules and the actual response data (solid black line).
